# Evaluation of Large Language Models for Peer Review in Transplantation Research: Algorithm Validation Study

**DOI:** 10.2196/84322

**Published:** 2026-02-11

**Authors:** Selena Ming Shen, Zifu Wang, Krittika Paul, Meng-Hao Li, Xiao Huang, Naoru Koizumi

**Affiliations:** 1 Pine View School Osprey, FL United States; 2 Center for Geographic Analysis Faculty of Arts and Sciences Harvard University Cambridge, MA United States; 3 Monta Vista High School Cupertino, CA United States; 4 Center for Biomedical Science and Policy Schar School of Policy and Government George Mason University Fairfax, VA United States; 5 Department of Environmental Sciences College of Arts and Sciences Emory University DeKalb, GA United States

**Keywords:** peer review, large language models, transplantation, bias, prompt engineering, retrieval-augmented generation, scholarly publishing, artificial intelligence, AI

## Abstract

**Background:**

Peer review remains central to ensuring research quality, yet it is constrained by reviewer fatigue and human bias. The rapid rise in scientific publishing has worsened these challenges, prompting interest in whether large language models (LLMs) can support or improve the peer review process.

**Objective:**

This study aimed to address critical gaps in the use of LLMs for peer review of papers in the field of organ transplantation by (1) comparing the performance of 5 recent open-source LLMs; (2) evaluating the impact of author affiliations—prestigious, less prestigious, and none—on LLM review outcomes; and (3) examining the influence of prompt engineering strategies, including zero-shot prompting, few-shot prompting, tree of thoughts (ToT) prompting, and retrieval-augmented generation (RAG), on review decisions.

**Methods:**

A dataset of 200 transplantation papers published between 2024 and 2025 across 4 journal quartiles was evaluated using 5 state-of-the-art open-source LLMs (Llama 3.3, Mistral 7B, Gemma 2, DeepSeek r1-distill Qwen, and Qwen 2.5). The 4 prompting techniques (zero-shot prompting, few-shot prompting, ToT prompting, and RAG) were tested under multiple temperature settings. Models were instructed to categorize papers into quartiles. To assess fairness, each paper was evaluated 3 times: with no affiliation, a prestigious affiliation, and a less prestigious affiliation. Accuracy, decisions, runtime, and computing resource use were recorded. Chi-square tests and adjusted Pearson residuals were used to examine the presence of affiliation bias.

**Results:**

RAG with a temperature of 0.5 achieved the best overall performance (exact match accuracy: 0.35; loose match accuracy: 0.78). Across all models, LLMs frequently assigned manuscripts to quartile 2 and quartile 3 while avoiding extreme quartiles (quartile 1 and quartile 4). None of the models demonstrated affiliation bias, though Gemma 2 (*P*=.08) and Qwen 2.5 (*P*=.054) were substantially biased. Each model displayed unique “personalities” in quartile predictions, influencing consistency. Mistral had the highest exact match accuracy (0.35) despite having both the lowest average runtime (1246.378 seconds) and computing resource use (7 billion parameters). While accuracy was insufficient for independent review, LLMs showed value in supporting preliminary triage tasks.

**Conclusions:**

Current open-source LLMs are not reliable enough to replace human peer reviewers. The largely absent affiliation bias suggests potential advantages in fairness, but these benefits do not offset the low decision accuracy. Mistral demonstrated the greatest accuracy and computational efficiency, and RAG with a moderate temperature emerged as the most effective prompting strategy. If LLMs are used to assist in peer review, their outputs require nonnegotiable human supervision to ensure correct judgment and appropriate editorial decisions.

## Introduction

### Background

Peer review is perceived as essential to the assurance of research quality and legitimacy [1], but there is a growing body of literature that recognizes its shortcomings. One of the greatest challenges is reviewer fatigue: while publications have surged exponentially [2], the reviewer pool has not kept pace, leaving reviewers overburdened, unrecognized, and unpaid [3-5]. Another major issue is reviewer bias. Humans are inherently biased; their life experiences, thinking styles, workload pressures, emotional state, and cognitive capacity can impact a paper’s acceptance decision [1]. Previous research has established that affiliation bias, the tendency to perceive manuscripts from renowned authors as more accurate, affects human review when it is not double-blinded [6,7]. The industry’s paradigm of human peer review is untenable due to the current model’s unsustainability and human reviewers’ inherent inconsistency. These 2 issues underscore the dire need to restructure the peer review process.

A much-debated question is whether generative artificial intelligence (AI) can help address the persistent challenges of peer review [8-10]. Large language models (LLMs) are able to complete a myriad of natural language processing tasks and have been extensively applied across medicine [11-15] and scientific research [10,16-23]. These capabilities position LLMs to significantly reduce the burden on human reviewers. A study by Tran et al [24] estimated that LLMs could reduce the peer review workload by 65%. Additionally, because LLMs lack personal motives or connections, they may help mitigate human bias in the peer review process [25]. Overall, current evidence suggests that generative AI may play a critical role in the future of scholarly publishing.

However, the integration of LLMs into peer review must be approached with utmost caution. Their participation in peer review has drawn heavy scrutiny due to known limitations, including factual inaccuracies, outdated content [26,27], and difficulties in upholding rigorous academic standards [28]. Another major concern with LLMs is their propensity to amplify historical biases. Stokel-Walker and Van Noorden [29] explained that “this unreliability is baked into how LLMs are built,” as they are trained on enormous datasets that include misinformation, outdated knowledge, and societal biases. This approach facilitates task-specific fine-tuning but also risks propagating harmful biases, including stereotypes and misrepresentations, that disproportionately affect communities considered marginalized [30]. Notably, the presence of affiliation bias, the tendency to perceive manuscripts from renowned authors as more accurate, in LLMs is under-studied despite its great relevance to peer review.

The primary research question guiding this study is as follows: Can current open LLMs reliably and fairly predict the prestige tier of the likely publication venue for a given transplantation manuscript? To address this question, this study identified the optimal combination of prompt engineering techniques and temperature settings for quartile prediction and compared LLMs in terms of decision accuracy, fairness, and runtime. To assess LLMs’ performance on specialized content—a known limitation of LLMs [29]—we used exclusively transplantation papers, a relatively small and focused research field [31]. Finally, we investigated the presence of affiliation bias in LLMs using chi-square tests for independence and adjusted Pearson residuals.

### Related Work

#### Promise of LLMs in Peer Review

Debate continues about whether LLMs are capable of supporting peer review. Conroy [32] argues that “the naive act of asking an LLM directly to review a manuscript is likely to produce little value beyond summaries and copy-editing suggestions.” However, empirical studies show that LLMs are already being adopted in practice and may offer meaningful benefits. Liang et al [23] uncovered that up to 17% of recent AI conference peer reviews were written by LLMs [33].

In a different study, Liang et al [34] found that over half of users rated GPT-4–generated feedback as helpful or very helpful, and 82.4% rated it more beneficial than feedback from at least some human reviewers. In the same vein, Thakkar et al [10] found that LLM-generated review feedback was more specific and actionable, enhancing peer review quality. Beyond generating feedback, LLMs have been used to evaluate the human peer review process and successfully identify reviewers’ biases, such as affiliation, anchoring, and gender biases [7,35]. These findings suggest that LLMs could support certain fairness-oriented tasks within the review pipeline.

Despite the emerging relevance, there is little published data on the capabilities of current open-source LLMs in peer review. Additionally, no previous study of LLM-conducted peer review has attempted to compare different prompt engineering techniques, even though prompt type can significantly impact LLM efficacy for a given task [36]. Moreover, few studies have investigated LLMs in relatively low-volume research areas, such as transplantation [31], where limited available data may impair LLM performance. These are critical knowledge gaps, as considerable evidence points to an already widespread use of LLMs in peer review workflows [33,37].

#### Affiliation Bias of LLMs in Peer Review

The academic literature on peer review has revealed the presence of affiliation bias in open peer review [6,7,38]. Biases are often rooted in reviewers’ personal experiences, connections, and beliefs, suggesting LLMs could potentially mitigate them in peer review. However, there are relatively few historical studies in the area of affiliation bias in LLMs in peer review. A study by von Wedel et al [25] offers a detailed analysis of affiliation bias in LLM peer review. Thirty preprint abstracts were combined with 30 affiliations and were provided to OpenAI’s GPT-3.5 for acceptance or rejection. The study found that higher-tiered affiliations were marginally associated with higher acceptance rates. Strikingly, differences during LLM peer review appeared to be smaller than those in previous reports on human affiliation bias (1.7% difference in LLM acceptance rates vs 12.5% difference in human acceptance rates), suggesting that LLMs reduce affiliation bias to a negligible amount.

However, the generalizability of this research was significantly constrained by several methodological limitations. The evaluation relied on a single LLM, raising questions about the applicability of the findings to the numerous available models. Furthermore, the token limit, which only allowed review of abstracts, likely diminished the relevance of the results to real-world scenarios involving full manuscript review. Finally, the dichotomous decision of acceptance or rejection did not consider the nuances of the scientific publishing ecosystem, particularly variations based on journal prestige.

### Current Challenges and Contributions of This Study

Under-studied aspects of LLM use in paper review include (1) comparison of the most recent open-source LLMs, (2) effects of different prompt engineering techniques on LLM decisions, (3) LLM review in the specialized field of transplantation, and (4) amplification of affiliation bias.

To address the current challenges with LLMs, this study concentrated on (1) assessing similarities and differences between several influential recent open-source LLMs [39] (eg, Meta’s Llama 3.3, Mistral AI’s Mistral, Google’s Gemma 2, DeepSeek r1-distill Qwen, and Alibaba’s Qwen 2.5); (2) assessing the effect of affiliations of varying prestige on the LLMs’ output; and (3) evaluating LLM accuracy using several common prompt engineering techniques (eg, zero-shot prompting, few-shot prompting, tree of thoughts [ToT] prompting, and retrieval-augmented generation [RAG]).

### Data Sources

The data collected and used in this study included journal articles published in the field of transplantation between 2024 and 2025. The journals were gathered from SCImago Journal and Country Rank and were separated into 4 quartiles based on rankings; each quartile represent the 25% of journals, with the first representing the top 25% of journals and the fourth representing the bottom 25% of journals [40]. Journal quartiles serve as tools to assess the quality and impact of academic journals [41].

A total of 200 papers were gathered, with 50 (25%) articles from each quartile. Each dataset entry included the attributes title, authors, publication date, journal name, journal abstract, full paper, and respective quartile (refer to Multimedia Appendix 1 for example data). The quartile and journal name were hidden when the data were input into the LLM but were later compared with LLM decisions to determine model accuracy.

## Methods

### Overview

**Figure 1:**
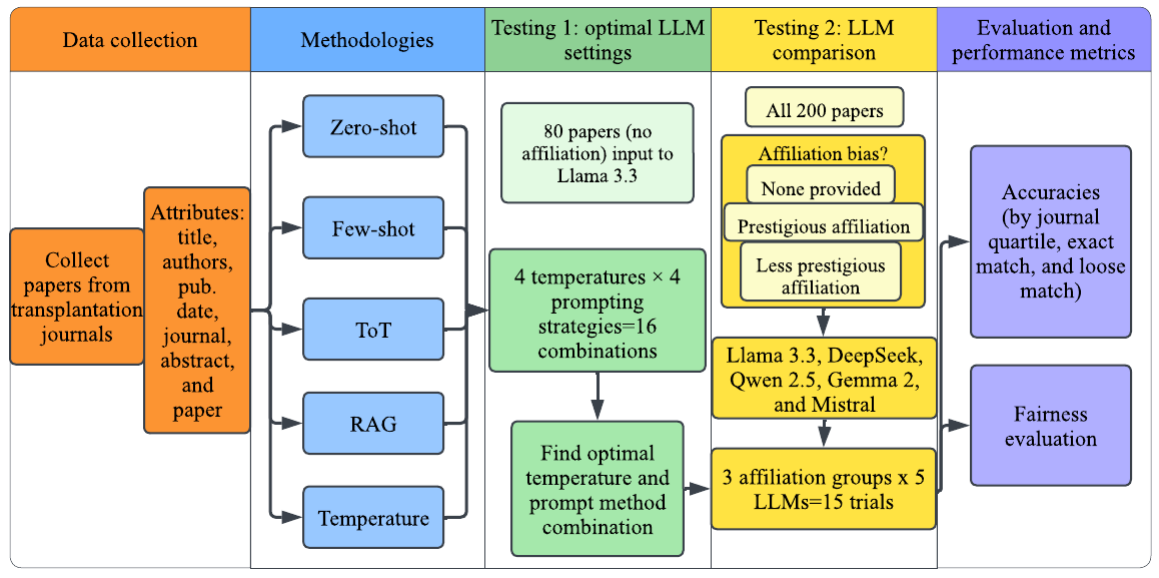
Workflow of investigating the presence of affiliation bias and capabilities for peer review in large language models (LLMs). RAG: retrieval-augmented generation; ToT: tree of thoughts.

After collecting 200 recent transplant publications, each paper was processed using 4 temperatures and 4 methods: zero-shot prompting, few-shot prompting, ToT prompting, and RAG. To make a more nuanced assessment of LLM decisions, the LLMs were given 4 options (quartile 1 [Q1], quartile 2 [Q2], quartile 3 [Q3], and quartile 4 [Q4]) rather than just 2 (acceptance vs rejection). Thus, all prompting methods were tuned with prompts to assign papers to journal quartiles. RAG was implemented using the open-source library Facebook AI Similarity Search, which was used to create a vector database for each paper [42]. The first round of testing was conducted using Llama 3.3 and 80 (40%) randomly sampled papers to identify a prompt-temperature combination that produced the highest accuracy (refer to Multimedia Appendix 2 for full prompts). This step served as a pilot hyperparameter search to determine optimal evaluation conditions. The same settings were then applied in round 2 testing, in which 5 open-source LLMs evaluated 200 (100%) papers across 3 trials: no affiliation, a prestigious affiliation, and a less prestigious affiliation. Finally, a chi-square test for independence was performed to detect whether there is an association between perceived affiliation and journal quartile. Effect sizes for associations were quantified using Cramer V, with 95% CIs calculated via nonparametric bootstrapping (5000 resamples). Accuracy scores, fairness, runtime, and computing resource use were used to compare the LLMs. This process is illustrated in Figure 1.

Journal quartiles were used as the prediction target. Although journal-level metrics are imperfect indicators of article-level quality, prior research consistently shows that they capture meaningful, though modest, signals. Thelwall et al [43] found that the correlation between article quality and journal impact was positive, with correlations around 0.4 in medicine. This aligns with broader bibliometric evidence that journals accumulate prestige largely because they tend to publish higher-quality or more influential work [44].

Given these empirical associations, quartiles offer a practical and reproducible proxy for the relative quality of a manuscript. Due to the lack of access to rejected or under review manuscripts, a binary accept-reject framework would have been uninformative; all papers in our dataset had already been accepted. Quartile prediction provided a more discriminative and challenging task, allowed the detection of model tendencies, and offered a standardized target that enabled controlled comparison across prompting strategies.

### Methods Configuration

Refer to Multimedia Appendix 2 for specific models tested, prompt templates used, and the RAG methodology.

#### Zero-Shot Prompting

In the zero-shot prompting configuration, the model was instructed to assign the given papers to journal quartiles without any additional examples to steer it.

#### Few-Shot Prompting

Few-shot prompting is a technique that enables in-context learning using demonstrations in the prompt to steer the model to better performance [45]. In the few-shot prompting configuration, an example system prompt, a provided paper, and an example response were provided. The system prompt clearly instructed the LLM to categorize given papers “in a consistent style.”

#### ToT Prompting

In ToT prompting, the LLM was encouraged to maintain a literal “tree of thoughts,” where thoughts represent coherent language sequences that serve as intermediate steps toward solving a problem. The LLM self-evaluated the progress made toward solving a problem through a deliberate reasoning process [45].

#### RAG Approach

RAG addressed LLM challenges, such as hallucination and outmoded knowledge, by retrieving external knowledge sources to complete tasks in addition to LLM’s static dataset. This was done through a built-in retrieval component that feeds relevant documents along with the prompt to the LLM [45]. The LLM was effectively fine-tuned to peer review without the need to retrain. RAG was the only prompting strategy capable of ingesting full papers; all other methods were restricted to abstracts due to token limitations.

#### Temperature

The temperature hyperparameter of an LLM regulates the amount of randomness in its response. On a scale of 0 to 1, a higher temperature results in more diverse or novel outputs, while a lower temperature results in more predictable or less creative outputs. A systematic grid search over 4 temperatures (0, 0.1, 0.5, and 1.0) was conducted with each prompting strategy. These values were selected to span the practical range from near-deterministic (0, 0.1) to moderately stochastic (0.5) to maximally stochastic (1.0), enabling observation of whether classification performance improved with more or less sampling diversity.

### LLM Comparison Metrics

To evaluate the performance of the LLMs in peer review, accuracy scores were calculated. Because LLMs have been found to give passable decisions but rarely give completely correct decisions [26], both “exact match” and “loose match” accuracies were calculated. Exact match accuracy is based on completely correct predictions of journal quartiles. For loose match accuracy, a prediction within 1 quartile of the input paper’s true quartile was considered correct. Finally, accuracy breakdowns per journal quartile were calculated to allow for further analysis of LLM behaviors. In addition, runtime and computing resource cost were compared to provide further insights into whether LLM performance was related to the amount of resources consumed.

### Fairness Evaluation

Each of the 5 LLMs evaluated the 200 collected transplantation papers 3 times: with no affiliation, artificial high-tier affiliation, and artificial low-tier affiliation. The high-tier and low-tier affiliations were chosen based on the Webometrics University Ranking of the number of citations amassed by research institutions in the last 6 years, with the National Institute of Health ranking first and the Walter Reed Army Institute ranking last. The website ceased to function after experimentation was completed, so it is not accessible, although a preprint describing it is available [46]. They were used to determine prestigious and less prestigious affiliations, respectively. A chi-square test for independence was used to test for association between affiliation and quartile. To quantify the strength of any observed association, effect sizes were calculated using Cramer V, with 95% CIs derived via nonparametric bootstrapping (5000 resamples). Results were considered statistically significant at an α level of .05. Adjusted Pearson residuals were also calculated, with residuals with an absolute value greater than 1.96 considered statistically significant [47]. Combined residuals for the top 2 and bottom 2 quartiles were also calculated for better interpretability, considering Q1 and Q2 as high-tier decisions and Q3 and Q4 as low-tier decisions.

### Ethical Considerations

This research did not require institutional review board approval because it did not meet the regulatory definition of human subjects research. The analysis was limited to publicly available literature and did not involve human participants, patient data, or identifiable personal information. According to 45 Code of Federal Regulations Part 46, such activities fall outside the scope of mandatory institutional review board approval.

## Results

### Optimal Configuration

**Figure 2:**
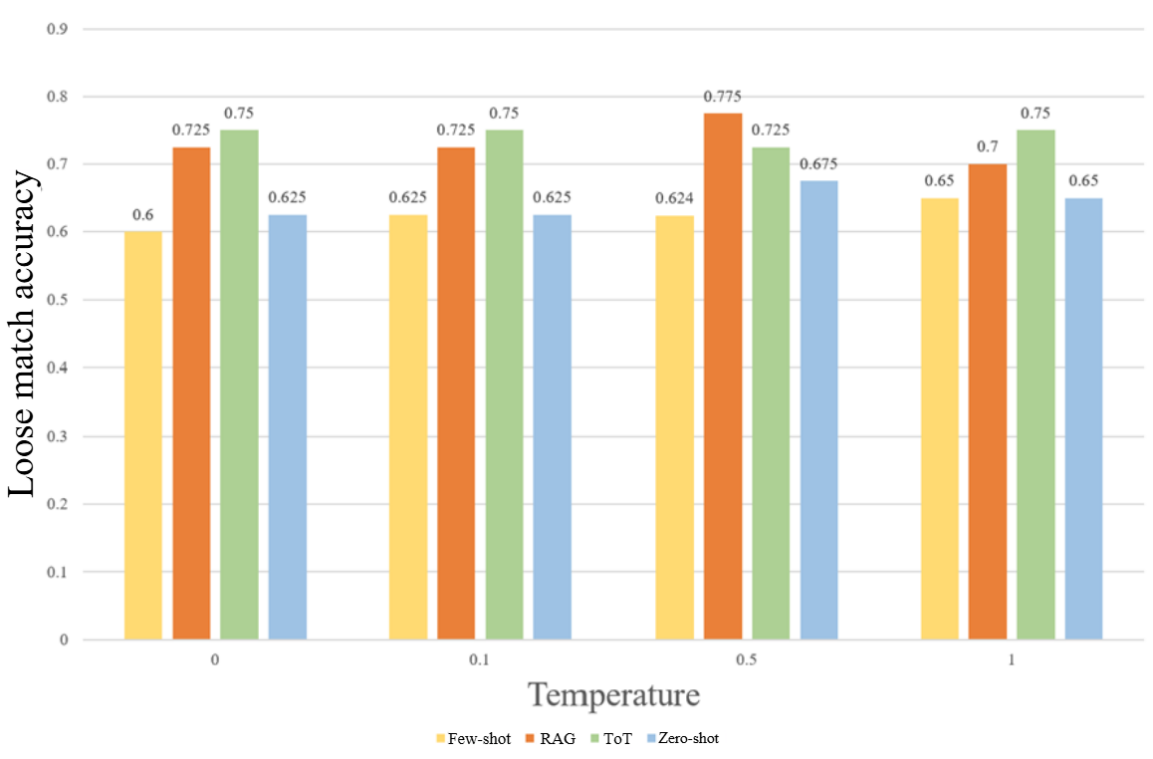
Loose match accuracies of combinations of temperature and prompt engineering techniques. RAG: retrieval-augmented generation; ToT: tree of thoughts.

As shown in Table 1 and Figure 2, the combination of RAG and a temperature of 0.5 yielded the highest exact match and loose match accuracies—0.35 and 0.775, respectively. RAG offered the most diversity in quartile predictions, while the other 3 strategies had 0% Q3 and Q4 accuracies. The zero-shot strategy resulted in the longest runtime and an overprediction of Q1. Few-shot prompting generally resulted in Q1 and Q2 decisions, though the results changed drastically with different temperatures, achieving the lowest exact match accuracy in Table 1 (0.2)—performing worse than random guessing. ToT drastically decreased LLM runtime and resulted in 100% Q2 accuracy, suggesting that the use of intermediate reasoning steps biased the model toward the neutral Q2 decision. Overall, exact match accuracies were extremely low. The difference in accuracies between Q1-Q2 and Q3-Q4 suggests the LLMs predicted the top 2 quartiles more frequently than the bottom 2, which corroborates previous studies’ findings that LLMs tend to inflate acceptance results [48,49].

These results illustrate the enhanced proficiency of RAG in peer review compared with other prompting methods. RAG possesses several advantages: retrieval of relevant information to improve model accuracy, greater response diversity, and a large context window that allows the LLM to read full papers [45,50]. RAG was the only prompting strategy capable of ingesting full papers, whereas all other methods were restricted to abstracts due to token limitations. Although this input-length asymmetry likely contributed to RAG’s superior performance, it also mirrors real-world deployment constraints, where many LLMs cannot natively process long scientific texts without retrieval augmentation. Thus, the comparison reflects each method’s practically usable form rather than an artificially equalized setting. The fundamental benefits RAG possesses over the other methods allow greater generalizability to the peer review process.

Interestingly, the most suitable temperature was 0.5 rather than lower temperatures, which were initially considered more suitable for the objective, fact-based peer review process. The temperature setting of 0.5 may strike a favorable balance between the objectivity and creativity required for review tasks.

**Table 1:** Comparative analysis of various large language model prompt engineering techniques under different temperature hyperparameters.

		Runtime (seconds)	Exact match accuracy (95% CI)	Loose match accuracy	Q1^a^ accuracy	Q2^b^ accuracy	Q3^c^ accuracy	Q4^d^ accuracy	
**RAG^e^** **with zero-shot prompting**									
	Temperature=1.0	1106.869	0.3 (0.21-0.41)	0.7	0.4	0.6	0.2	0.0	
	Temperature=0.5	1089.631	*0.35*^f^ (0.25-0.46)	*0.775* ^f^	0.5	0.7	0.2	0.0	
	Temperature=0.1	1109.433	0.325 (0.23-0.43)	0.3	0.4	0.7	0.2	0.0	
	Temperature=0	1107.765	0.3 (0.21-0.41)	0.275	0.4	0.6	0.2	0.0	
**Zero-shot prompting**									
	Temperature=1.0	1821.130	0.275 (0.19-0.38)	0.65	0.8	0.3	0.0	0.0	
	Temperature=0.5	1814.739	0.325 (0.23-0.41)	0.675	0.9	0.4	0.0	0.0	
	Temperature=0.1	1824.464	0.3 (0.21-0.41)	0.625	0.8	0.4	0.0	0.0	
	Temperature=0	1832.114	0.3 (0.21-0.41)	0.625	0.8	0.4	0.0	0.0	
**Few-shot prompting**									
	Temperature=1.0	1743.013	0.25 (0.20-0.31)	0.65	0.6	0.4	0.0	0.0	
	Temperature=0.5	1737.973	0.325 (0.23-0.43)	0.624	0.9	0.4	0.0	0.0	
	Temperature=0.1	1729.847	0.2 (0.15-0.26)	0.625	0.4	0.4	0.0	0.0	
	Temperature=0	1755.463	0.275 (0.19-0.38)	0.6	0.7	0.4	0.0	0.0	
**Tree of thoughts prompting**									
	Temperature=1.0	615.322	0.3 (0.21-0.41)	0.75	0.2	1.0	0.0	0.0	
	Temperature=0.5	596.043	0.275 (0.19-0.38)	0.725	0.1	1.0	0.0	0.0	
	Temperature=0.1	613.613	0.25 (0.20-0.31)	0.75	0.0	1.0	0.0	0.0	
	Temperature=0	615.121	0.25 (0.20-0.31)	0.75	0.0	1.0	0.0	0.0	

^a^Q1: quartile 1.

^b^Q2: quartile 2.

^c^Q3: quartile 3.

^d^Q4: quartile 4.

^e^RAG: retrieval-augmented generation.

^f^Italics indicate the highest accuracy.

### Fairness

Table 2 presents the quartile decisions across the 3 different input affiliations, along with the *P* values of the chi-square test for independence and the corresponding effect sizes (Cramer V) with 95% CIs. The *P* values of Gemma 2 and Qwen 2.5 are approximately an order of magnitude smaller than the other *P* values, demonstrating greater statistical significance. However, the effect sizes for all models were negligible (Cramer V≤0.10), with CIs indicating that any true association between affiliation and quartile decision was minimal.

To break down the results of this test, adjusted Pearson residuals are presented in Table 3, where positive values indicate overrepresentation and negative values indicate underrepresentation relative to expected response frequencies. One unanticipated result is that Gemma significantly overpredicted the number of Q4 papers when given no affiliation. By contrast, when given no affiliation, Qwen significantly overpredicted the number of Q1 papers. Interestingly, when given a prestigious affiliation, Qwen placed more papers in Q4. These statistically significant associations did not align with affiliation bias, as that would entail overestimating Q1 and Q2 and underestimating Q3 and Q4 decisions when given prestigious affiliations.

Overall, none of the LLMs exhibited affiliation bias.

**Table 2:** Model quartile decisions across input affiliations, chi-square test for independence *P* values, effect sizes, and 95% CIs.

Models and affiliation level			Q1^a^, 50 (25%)		Q2^b^, 50 (25%)		Q3^c^, 50 (25%)		Q4^d^, 50 (25%)		*P* value			Cramer V (95% CI)	
**Llama 3.3-70B**												.80			0.05083 (0.047-0.127)
	None	37		148		15		0							
	High tier	48		139		12		1							
	Low tier	41		144		14		1							
**Mistral-7B**												.63			0.04621 (0.027-0.120)
	None	54		101		45		0							
	High tier	55		112		33		0							
	Low tier	54		109		37		0							
**Gemma 2-9B**												*.08* ^e^			0.08408 (0.045-0.153)
	None	0		24		157		19							
	High tier	0		27		166		7							
	Low tier	0		24		168		8							
**DeepSeek r1-distill Qwen-14B**												.87			0.04516 (0.041-0.123)
	None	3		73		113		11							
	High tier	1		83		103		13							
	Low tier	3		79		106		12							
**Qwen 2.5-7B**												*.05*			0.10159 (0.071-0.161)
	None	6		51		143		0							
	High tier	2		69		127		2							
	Low tier	1		60		139		0							

^a^Q1: quartile 1.

^b^Q2: quartile 2.

^c^Q3: quartile 3.

^d^Q4: quartile 4.

^e^Italicization indicates relatively significant *P* values.

**Table 3:** Adjusted Pearson residuals of large language model decisions.

Models and affiliation level			Q1^a^ (adjusted Pearson residuals)		Q2^b^ (adjusted Pearson residuals)		Q3^c^ (adjusted Pearson residuals)		Q4^d^ (adjusted Pearson residuals)		Q1 and Q2 (adjusted Pearson residuals)		Q3 and Q4 (adjusted Pearson residuals)
**Llama 3.3-70B**													
	None	−1.063		0.8343		0.4576		−1.0017		−0.2288		−0.5440	
	High tier	11.2757		−0.8985		−0.5720		0.5008		0.3773		−0.0712	
	Low tier	−0.2126		0.0642		0.1144		0.5008		−0.1484		0.6152	
**Mistral-7B**													
	None	−0.0649		−1.0999		1.4668		0		−1.1648		1.4668	
	High tier	0.1298		0.8105		−1.1734		0		0.9403		−1.1734	
	Low tier	−0.0649		0.2895		−0.2934		0		0.2246		−0.2934	
**Gemma 2-9B**													
	None	0		−0.2619		−1.4974		*2.8717* ^e^		−0.2619		1.3743	
	High tier	0		0.5237		0.5240		−1.6231		0.5237		−1.0991	
	Low tier	0		−0.2619		0.9732		−1.2486		−0.2619		−0.2753	
**DeepSeek r1-distill Qwen-14B**													
	None	0.5377		−0.9462		0.9841		−0.3647		−0.4086		0.6195	
	High tier	−1.0753		0.8280		−0.7526		0.3647		−0.2474		−0.3879	
	Low tier	0.5377		0.1183		−0.2316		0		0.6559		−0.2316	
**Qwen 2.5-7B**													
	None	*2.1374*		−1.7008		1.2394		−1.0017		0.4366		0.2377	
	High tier	−0.7125		1.7008		−1.7352		*2.0033*		0.9884		0.2682	
	Low tier	−1.4249		0		0.4958		−1.0017		−1.4249		−0.5059	

^a^Q1: quartile 1.

^b^Q2: quartile 2.

^c^Q3: quartile 3.

^d^Q4: quartile 4.

^e^Italicization indicates statistically significant residuals.

### Model Comparisons

**Figure 3:**
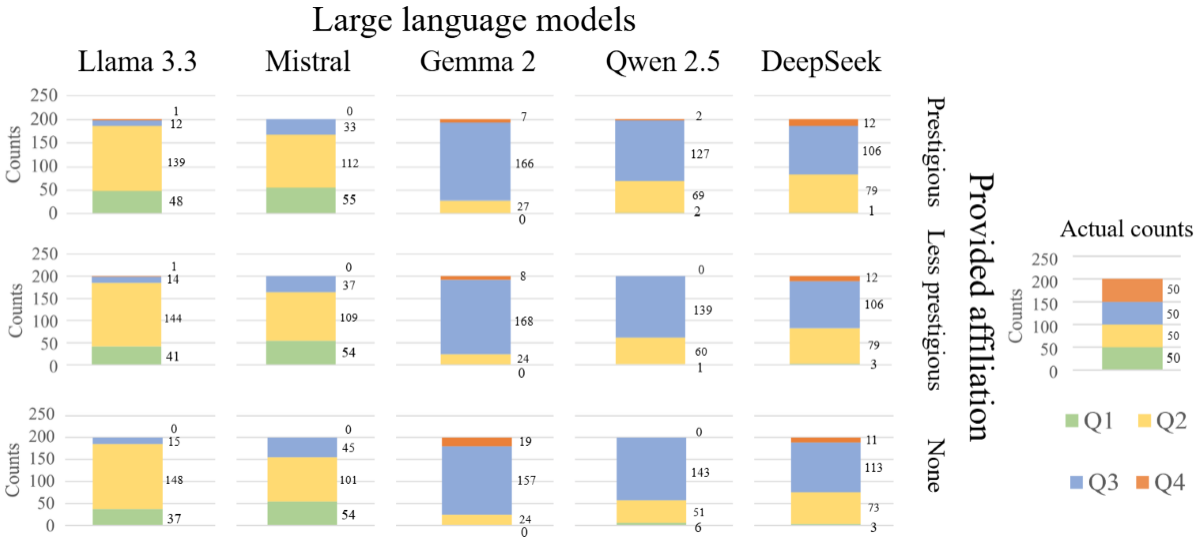
Distribution of large language model decisions across the provided affiliations. Q1: quartile 1; Q2: quartile 2; Q3: quartile 3; Q4: quartile 4.

In the second round of testing, Mistral 7B had the highest exact match accuracy, and Qwen 2.5 had the highest loose match accuracy among the LLMs (Table 4). Notably, each LLM had a unique “personality” or preference for the quartiles in which it placed papers. As shown in Figure 3, Llama 3.3 preferred Q2 followed by Q1, Mistral preferred Q2 closely followed by Q1, Gemma preferred Q3 followed by Q2, DeepSeek preferred Q3 followed by Q2, and Qwen preferred Q3 followed by Q2. The LLMs exhibited low overall accuracy, which was expected, as they were not trained for technical topics [17]. A previous study found that LLMs tend to be biased toward technical excellence over the novelty of submitted experiments [51]. Interestingly, the LLMs tended to avoid placing papers in either extreme quartile (Q1 or Q4).

Comparisons of LLM runtimes and sizes are provided in Multimedia Appendix 3. Qwen 2.5 had the greatest average runtime by far (7248.741 seconds), though it also had the greatest loose match accuracy. Llama 3.3 had the second greatest average runtime (1325.805 seconds) and the largest size by a significant margin (70B). Mistral had the lowest average runtime (1246.378 seconds) and the smallest size (7B), yet it impressively achieved the highest exact match accuracy.

**Table 4:** Runtime and accuracy of model predictions.

Run			Runtime (seconds)		Exact match accuracy (95% CI)		Loose match accuracy		Q1^a^ accuracy		Q2^b^ accuracy		Q3^c^ accuracy		Q4^d^ accuracy
**Llama 3.3-70B**															
	Run 1	1331.919		0.275 (0.227-0.297)		0.755		0.16		0.86		0.08		0	
	Run 2	1318.557		0.25 (0.227-0.297)		0.77		0.22		0.76		0.02		0	
	Run 3	1326.938		0.255 (0.227-0.297)		0.765		0.26		0.74		0.02		0	
**Mistral-7B**															
	Run 1	1245.114		0.295 (0.284-0.358)		0.785		0.46		0.52		0.2		0	
	Run 2	1249.270		*0.35 (0.284-0.358)* ^e^		0.775		0.52		0.64		0.24		0	
	Run 3	1244.751		0.315 (0.284-0.358)		0.76		0.38		0.64		0.24		0	
**Gemma 2-9B**															
	Run 1	1250.046		0.255 (0.257-0.329)		0.77		0.06		0.26		0.7		0	
	Run 2	1439.940		0.315 (0.257-0.329)		0.82		0		0.46		0.76		0.04	
	Run 3	1229.739		0.305 (0.257-0.329)		0.795		0		0.42		0.8		0	
**DeepSeek r1–distill Qwen-14B**															
	Run 1	1317.195		0.28 (0.252-0.324)		0.81		0		0.06		0.82		0.24	
	Run 2	1309.082		0.27 (0.252-0.324)		0.815		0		0.14		0.9		0.04	
	Run 3	1257.635		0.31 (0.252-0.324)		0.8		0		0.22		0.94		0.08	
**Qwen 2.5-7B**															
	Run 1	7211.855		0.296 (0.270-0.343)		0.835		0		0.48		0.68		0.02	
	Run 2	7302.680		0.315 (0.270-0.343)		*0.84*		0		0.44		0.74		0.08	
	Run 3	7231.687		0.305 (0.270-0.343)		0.825		0		0.56		0.64		0.02	

^a^Q1: quartile 1.

^b^Q2: quartile 2.

^c^Q3: quartile 3.

^d^Q4: quartile 4.

^e^Italicization indicates the highest accuracy.

## Discussion

### Principal Findings

Our findings can inform future efforts to use LLMs in peer review. The data indicated that while affiliation bias was not present in current LLMs, their prediction accuracy was insufficient to replace human reviewers at this time. Notably, the LLMs studied appeared to have distinct “personalities” that preferred to allocate papers to journals of a specific quartile. Across the board, the LLMs avoided allocating to the extremes of Q1 and Q4, instead preferring predictions in Q2 and Q3, corroborating previous studies’ findings that LLMs struggle to provide critical feedback comparable to human reviewers [11].

Our experimental design underscores 2 methods with outsized impact: knowledge retrieval and sampling diversity. RAG shattered the context-window ceiling that limited earlier studies to abstract-level inputs, enabling full-text appraisal that mirrors human practice.

Another surprising result arose from temperature tuning. We hypothesized that near-deterministic sampling (eg, temperature ≤0.2) would best serve an “objective task”; instead, a moderate temperature of 0.5 struck the sweet spot between rigidity and adaptability. This finding aligns with emerging cognitive science analogies that liken temperature to divergent thinking in human creativity: too low and the model becomes dogmatic, too high and it hallucinates. A logical extension is adaptive temperature schedules, where the model introspects and modulates creativity as necessary—high creativity for speculative synthesis and low for citation verification.

A key result is the near eradication of affiliation bias. In historical datasets, manuscripts bearing a globally recognizable university crest enjoy acceptance advantages of roughly 1 in 8 decisions [6]; in our LLM trials, models consistently predicted in Q2 and Q3. Llama 3.3 and Mistral often chose to place papers in Q2, while Gemma 2, DeepSeek, and Qwen 2.5 more often chose Q3. At first glance, this is cause for celebration: a plausible pathway toward a peer review ecosystem that rewards intellectual merit rather than institutional pedigree. However, an equally important, if less comfortable, lesson is that “LLM objectivity” is conditional and brittle. Gemma 2 and Qwen 2.5 inched uncomfortably close to significance (*P*=.08 and *P*=.054, respectively), raising the possibility that model bias may reemerge as training corpora, instruction-tuning objectives, or deployment prompts shift over time.

Raw performance metrics make a compelling case for restraint. Exact match accuracy peaked at 35%, a level that would be untenable as the sole basis for publication decisions. Loose match accuracy reached 78%, but this metric is largely a reflection of centrist allocation: with most papers placed in Q2 or Q3, a large proportion fall within one quartile by default rather than due to genuine assessment of scientific merit. These results strongly suggest that current LLMs cannot replace human reviewers. LLMs should be restricted to acting as high-recall assistants that flag methodological red flags and assemble structured digests, while reserving nuanced judgment and field-specific contextualization for human experts.

Beyond headline accuracy, our study uncovered a subtler, systemic risk: each LLM exhibits a stable preference profile—a “personality” in editorial decisions. Llama 3.3 habitually gravitates toward Q2, while Mistral tends to give generous Q1 and Q2 ratings. By contrast, Qwen 2.5, DeepSeek, and Gemma 2 have the counterintuitive habit of demoting elite-affiliated papers to Q3—a behavior that human reviewers rarely exhibit. These anomalies likely originate deep in the pretraining soup of web pages, blogs, and archival documents where prestige cues intermix with polemics, conspiracies, and outdated citation networks. What appears as “objectivity” may instead be an averaging of contradictory signals rather than a principled neutrality. Crucially, these inherent biases could distort acceptance profiles if models are used in practice. Journals unaware of these quirks risk subtly penalizing high-quality work or promoting safe-but-unremarkable manuscripts. This serves as a warning: without careful monitoring, audits, and ongoing validation, reliance on LLMs for peer review decisions could unintentionally introduce new forms of systematic error rather than reduce bias.

### Limitations and Future Research

A central limitation is the use of journal quartiles as ground truth labels. Individual articles vary widely within the same journal, editorial decisions reflect a mixture of scientific and contextual factors, and journal prestige can correlate with stylistic conventions (writing density, terminology, and methodological templates) [52]. Quartiles offer a practical and standardized metric, but they imperfectly represent article-level quality and may lead LLMs to predict based on venue-specific stylistic cues rather than substantive scientific merit. Future studies should incorporate richer outcome labels, including a 5-option decision set (Q1, Q2, Q3, Q4, or not publishable), reviewer scores, or editorial decisions.

Additionally, because only published manuscripts were included, the dataset lacks the full spectrum of real-world submissions. Incorporating preprints would more closely approximate authentic peer review conditions.

Finally, while powerful, LLMs are known to be somewhat unstable: even when provided with identical prompts, an LLM’s outputs may be inconsistent in factual content (hallucinations) or in misinformation provided [53]. Future research on this topic may benefit from incorporating multiple trials for each LLM and prompt strategy to account for this variability to determine whether the patterns observed here hold across topics, disciplines, and manuscript types.

### Conclusions

This study (1) investigated the presence of affiliation bias in LLM peer review, (2) evaluated the proficiency of popular open-source LLMs in the prediction of journal quartiles for transplantation papers, and (3) determined the effect of different prompting methods and temperatures on LLM peer review. While the LLMs were found to be free of affiliation bias, they struggled to provide exact, correct answers. This highlights the limited capacity of current LLMs for autonomous peer review and the nonnegotiable need for human supervision if used. Finally, Mistral had the highest accuracy and efficiency among all the models, and RAG combined with a temperature of 0.5 was the best-performing combination of prompting methods, although by a small margin.

## Appendix

Multimedia Appendix 1 Example data source.

Multimedia Appendix 2 Experimental design and prompt materials.

Multimedia Appendix 3 Comparison of large language model runtimes and sizes.

## Abbreviations

AI: artificial intelligence
LLM: large language model
RAG: retrieval-augmented generation
ToT: tree of thoughts
Q1: quartile 1
Q2: quartile 2
Q3: quartile 3
Q4: quartile 4

## Data Availability

The datasets generated or analyzed during this study are available from the corresponding author on reasonable request.
