# Supplementary material for: Evaluation of Large Language Models for Peer Review in Transplantation Research: Algorithm Validation Study
Source: JMIR AI. 2026 Feb 11;5:e84322. doi: 10.2196/84322 (PMC12936655; doi:10.2196/84322)
Supplement: Multimedia Appendix 1 [file ai_v5i1e84322_app1.docx]

**Multimedia Appendix 1: Example Data Source**

This appendix provides the structure of the collected dataset, along with an example paper [1].

| **Attribute Title** | Increased Pretransplant Inflammatory Biomarkers Predict Death With Function After Kidney Transplantation |
| --- | --- |
| **Author(s)** | Lorenz, Elizabeth C.; Smith, Byron H.; Liang, Yun; Park, Walter D.; Bentall, Andrew J.; Dhala, Atiya F.; Waterman, Amy D.; Kennedy, Cassie C.; Hickson, LaTonya J.; Rule, Andrew D.; Cheville, Andrea L.; LeBrasseur, Nathan K. & Stegall, Mark D. |
| **Publication Date** | 24-Jun-24 |
| **Journal Name** | Transplantation |
| **Abstract** | "Chronic systemic inflammation is associated with mortality in patients with chronic kidney disease, cardiovascular disease, and diabetes. The goal of this study was to examine the relationship between pretransplant inflammatory biomarkers (growth differentiation factor-15 [GDF-15], interleukin-6 [IL-6], soluble tumor necrosis factor receptor-1, monokine induced by gamma interferon/chemokine [C-X-C motif] ligand 9 [MIG/CXCL9], monocyte chemoattractant protein-1, soluble FAS, tumor necrosis factor-α, interleukin-15, and interleukin-1β) and death with function (DWF) after kidney transplantation (KT). We retrospectively measured inflammatory biomarker levels in serum collected up to 1 y before KT (time from blood draw to KT was 130 ± 110 d) in recipients transplanted between January 2006 and December 2018...” |
| **Paper** | Kidney transplantation (KT) offers better quality of life and improved patient survival compared with dialysis. However, KT recipients experience up to a 6-fold higher mortality rate compared with the general population.1 In fact, in the first 10 y after KT, the most common cause of graft loss is death with a functioning (DWF) allograft.2 Thus, improving long-term survival of KT recipients is a major unmet need. Identifying novel contributors to DWF is an important strategy toward improving long-term KT survival.3,4  Chronic inflammation, defined as increased serum levels of inflammatory cytokines and related biomarkers (high-sensitivity C-reactive protein, fibrinogen, pro-inflammatory cytokines such as interleukin-15 [IL-15], interleukin-1β [IL-1β], interleukin-6 [IL-6], tumor necrosis factor-α [TNFα]), has emerged as a central mechanism of morbidity and mortality of many chronic diseases, including cardiovascular disease, diabetes, cancer, and kidney failure.5,6 Furthermore, the observation that chronic inflammation is common in older people has led to the concept of “inflammaging” that features chronic inflammation as an integral part of normal aging. However, chronic inflammation can occur in any age group—even children.7 In many instances, chronic inflammation is characterized by increased numbers of activated, mature lymphocytes and macrophages at sites of inflammation.8 Ironically, this chronically activated immune system may be dysfunctional leading to increased rates of infection and cancer.9-11 Additionally, chronic inflammation has been associated with cellular senescence, or irreversible cell cycle arrest, although it is unclear whether the senescent features are the cause and/or the result of chronic inflammation.9 These findings suggest that rather than a specific disease entity, chronic inflammation is likely a complex and dynamic mechanistic process contributing to both normal aging and chronic disease…” |

### References

1. Lorenz, Elizabeth C et al. “Increased Pretransplant Inflammatory Biomarkers Predict Death With Function After Kidney Transplantation.” *Transplantation* vol. 108,12 (2024): 2434-2445. doi:10.1097/TP.0000000000005103
