# Supplementary material for: Evaluation of Large Language Models for Peer Review in Transplantation Research: Algorithm Validation Study
Source: JMIR AI. 2026 Feb 11;5:e84322. doi: 10.2196/84322 (PMC12936655; doi:10.2196/84322)
Supplement: Multimedia Appendix 2 [file ai_v5i1e84322_app2.docx]

**Multimedia Appendix 2: Experimental Design and Prompt Materials**

This appendix provides the necessary materials to reproduce the experimental setup of the study, including specific models tested, prompts used, and RAG setup.

**Part 1: LLMs tested**

The following five models were downloaded and ran in LM Studio:


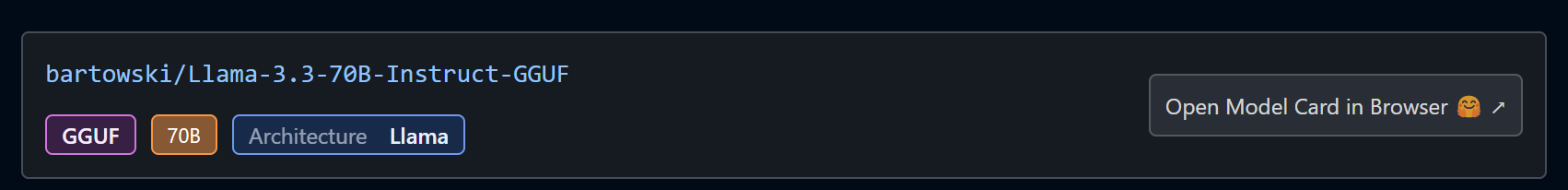


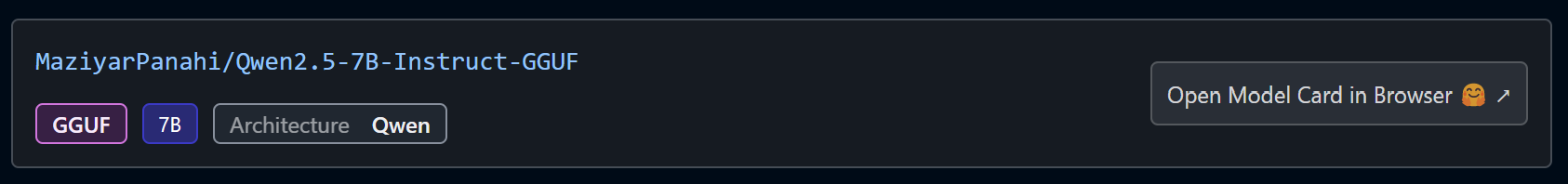


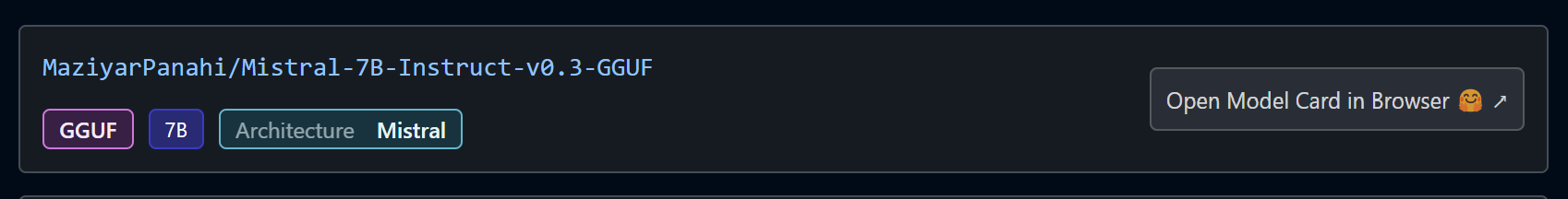


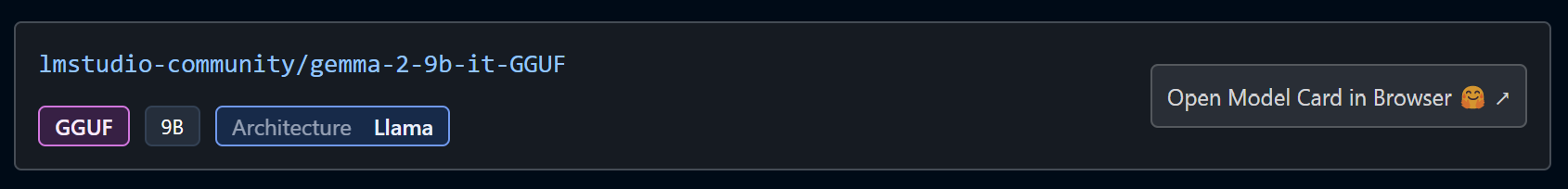


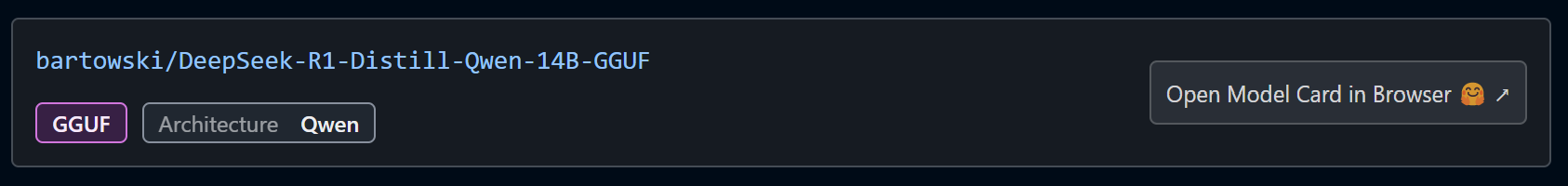


**Part 2: Prompt Templates**

The LLM’s cache was cleared after each input to stay within the context window limit.

| **Prompt Type** | Template |
| --- | --- |
| **Zero shot**  **(only abstract)** | “““You are a journal reviewer. Please determine if the given paper should be published in Q1, Q2, Q3, or Q4 journals. Use the following format:    Overall evaluation: <summary of paper in 1-2 sentences>  Strengths: <paper’s strengths>  Weaknesses: <paper’s weaknesses>  Decision: <journal quartile>  Reasoning: <reasoning for decision>  ””” |
| **Few shot**  **(only abstract)** | “”” You are a transplant journal reviewer that decides whether the following paper deserves to be accepted into a Q1, Q2, Q3, or Q4 journal. Do it in a consistent style.  <applicant>:  Here is my abstract: “Anemia is common after kidney transplantation. The etiology may be multifactorial, such as causes of anemia in the general population and causes that are unique to the kidney transplant setting. Posttransplant anemia, particularly when severe, may be associated with adverse effects such as graft failure, mortality, and a decline in kidney function. After careful investigation, that is, having excluded or treated reversible causes of anemia, treatment of anemia in patients with a kidney transplant is based on iron supplementation or erythropoiesis-stimulating agents (ESA), although there are no specific guidelines on anemia management in this patient population. Iron therapy is often needed, but optimal and safe iron-deficiency management strategies remain to be defined. Evidence suggests that ESAs are safe and potentially associated with favorable outcomes. Better graft function has been reported with ESA use targeting hemoglobin levels higher than those recommended in the general population with chronic kidney disease and with no apparent increased risk of cardiovascular events. These results require further investigation. Data on the use of hypoxia-inducible factor inhibitors are limited. Prevention and treatment of anemia in kidney transplantation can improve patients’ quality of life, life expectancy, allograft function, and survival.”  <transplant journal reviewer>:  “Overall Evaluation: This paper provides a comprehensive review of the etiology, diagnosis, and management of anemia in kidney transplant recipients. The authors discuss the unique challenges and potential treatments for post-transplant anemia, focusing on iron therapy and erythropoiesis-stimulating agents.  Strengths:  1. The paper presents a clear understanding of the multifactorial causes of anemia in kidney transplant recipients and highlights its clinical significance.  2. It discusses the rationale for iron supplementation and ESA use, providing evidence from various studies that support their use.  3. The authors emphasize the importance of addressing anemia to improve patients' quality of life, allograft function, and survival.  Weaknesses:  1. Some sections lack specific data or references to strengthen the claims made.  2. The discussion on hypoxia-inducible factor inhibitors is brief and could benefit from more research and data.  Decision: Q1  Reasoning: Despite minor weaknesses, this paper offers a well-rounded understanding of anemia management in kidney transplant recipients. It provides valuable insights into the challenges faced in this patient population and discusses potential treatment strategies that could improve patient outcomes. Further research is needed to define optimal iron-deficiency management strategies and explore the use of hypoxia-inducible factor inhibitors in kidney transplant anemia.”  <applicant>:  “Here is my abstract: <actual abstract>”  <transplant journal reviewer>:  ””” |
| **Tree of Thought**  **(only abstract)** | “”” Imagine three different experts are answering this question./ All experts will write down 1 step of their thinking,/ then share it with the group./ Then all experts will go on to the next step, etc./ If any expert realizes they're wrong at any point, then they leave./ The question is which journal would this paper be accepted into: Q1, Q2, Q3, or Q4?/ The steps they go through are 1) overall evaluation, 2) strengths, 3) weaknesses, 4) decision, 5) decision reasoning./ Please print the answers the experts agreed on for each of the steps./  Use the following format:  Overall evaluation: <summary of paper in 1-2 sentences>  Strengths: <paper’s strengths>  Weaknesses: <paper’s weaknesses>  Decision: <journal quartile>  Reasoning: <reasoning for decision>  “”” |
| **RAG**  **(both abstract and full paper)** | “““You are a journal reviewer. Please determine if the given paper should be published in Q1, Q2, Q3, or Q4 journals. Use the following format:    Overall evaluation: <summary of paper in 1-2 sentences>  Strengths: <paper’s strengths>  Weaknesses: <paper’s weaknesses>  Decision: <journal quartile>  Reasoning: <reasoning for decision>  ””” |

**Part 3: RAG setup**

We implemented the RAG approach using LlamaIndex and a FAISS vector store. The retrieval corpus consisted of the full texts of all 200 papers collected for this study. Each paper was treated as a single chunk, without additional segmentation. We computed vector embeddings using the nomic-embed-text-v1.5 model (embedding dimension = 768) and stored the resulting vectors in a FAISS IndexFlatL2 index. Similarity search between the query and all document vectors was performed using L2 distance. For query construction and retrieval, we embedded the same zero-shot reviewer-style prompt described above and set similarity_top_k = 1 to retrieve the most relevant paper from the index. The LLM was then provided with both the query and the retrieved document, which served as contextual reinforcement, to generate the final predicted journal-quartile classification.
