# Supplementary figures and images for: Evaluation of Large Language Models for Peer Review in Transplantation Research: Algorithm Validation Study
Source: JMIR AI. 2026 Feb 11;5:e84322. doi: 10.2196/84322 (PMC12936655; doi:10.2196/84322)

**Multimedia Appendix 3: Comparison of LLM runtimes and sizes**

**
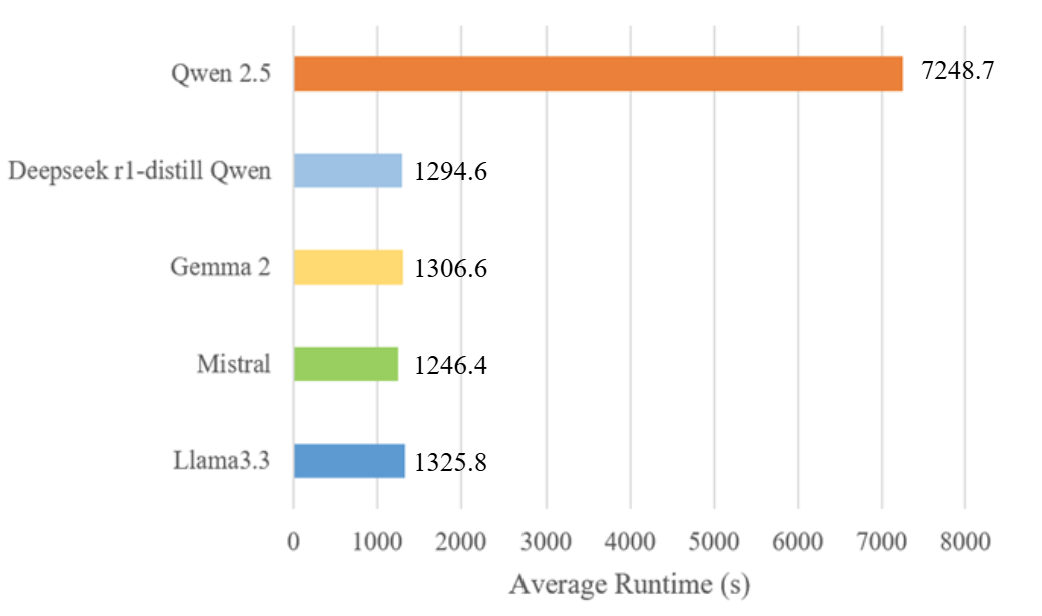
**

**
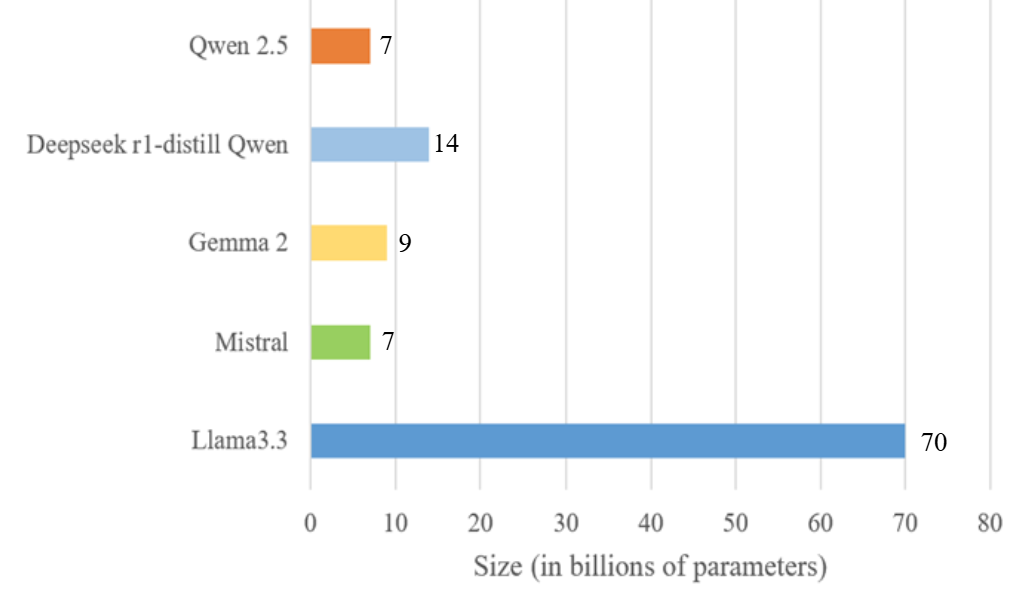
**

Supplement: Multimedia Appendix 3 [file ai_v5i1e84322_app3.docx]
